# Is improvement in depression in patients attending cardiac rehabilitation with new-onset depressive symptoms determined by patient characteristics?

**DOI:** 10.1136/openhrt-2020-001264

**Published:** 2020-08-26

**Authors:** Serdar Sever, Patrick Doherty, Su Golder, Alexander Stephen Harrison

**Affiliations:** Department of Health Sciences, University of York, York, North Yorkshire, UK

**Keywords:** cardiac rehabilitation, depression, coronary artery disease

## Abstract

**Background:**

Patients with cardiovascular disease (CVD) commonly experience depressive symptoms which is associated with adverse outcome and increased mortality. Examining the baseline characteristics of cardiac rehabilitation (CR) patients that determine Hospital Anxiety and Depression Scale (HADS) depression outcome may facilitate adjustments in CR programme delivery. This study aims to investigate whether comorbidities, demographic and clinical characteristics of patients, with new-onset post-cardiac event depressive symptoms, determine change in their depression following CR.

**Methods:**

Analysing the routine practice data of British Heart Foundation National Audit of Cardiac Rehabilitation between April 2012 and March 2018, an observational study was conducted. Patients with new-onset post-cardiac event depressive symptoms and no previous documented history of depression constituted the study population.

**Results:**

The analyses included 64 658 CR patients (66.24±10.69 years, 75% male) with new-onset HADS measures, excluding patients with a history of depression. The comorbidities determining reduced likelihood of improvement in depression outcomes after CR were angina, diabetes, stroke, emphysema and chronic back problems. In addition, higher total number of comorbidities, increased weight, a higher HADS anxiety score, smoking at baseline, physical inactivity, presence of heart failure and being single were other significant determinants. However, receiving coronary artery bypass graft treatment was associated with better improvement.

**Conclusion:**

The study identified specific baseline comorbid conditions of patients with new-onset depressive symptoms including angina, diabetes, stroke, emphysema and chronic back problems that were determinants of poorer mental health outcomes (HADS) following CR. Higher total number of comorbidities, increased weight, physical inactivity, smoking, presence of heart failure and being single were other determinants of a negative change in depression. These findings could help CR programmes focus on tailoring the CR intervention around comorbidity, physical activity status, weight management and smoking cessation in patients with new-onset depressive symptoms.

**Key questions**

**What is already known about this subject?**

- Patients with cardiovascular disease often experience depressive symptoms that is associated with increased mortality and adverse outcomes. However, there is a lack of studies which investigate baseline characteristics of cardiac rehabilitation (CR) patients that determine depression outcome in patients with new-onset depressive symptoms.

**What does this study add?**

- The current study is the first to examine the patient characteristics that determine the change in depressive symptoms following CR, specifically in patients with new-onset post-cardiac event depressive symptoms.

**How might this impact on clinical practice?**

- This paper shows that patients with new-onset depressive symptoms are less likely to improve their depression levels following CR when they have such comorbidities and patient characteristics at baseline: comorbidities of angina, diabetes, stroke, emphysema, chronic back problems, and higher total number of comorbidities, increased weight, physical inactivity, smoking, presence of heart failure and being single according to multivariate analysis results. These findings could enable CR programmes to tailor the CR intervention around comorbidities, physical activity status, weight management and smoking cessation in this specific patient population.

## Introduction

The association of depression with increased mortality is widely demonstrated in patients with cardiovascular disease (CVD) in a previous meta-analysis^1 2^ as well as its association with poor cardiac prognosis. The American Heart Association recently published a scientific statement which recommends elevating depression to the status of a risk factor for mortality and cardiac morbidity in patients with CVD.^3^ Depression is a common condition, one in five of patients with CVD experience depression^4^ and it is one of the leading causes of years lived with disability globally.^5^ Depression is associated with loss of productivity and treatment non-compliance in patients with CVD,^6^ along with increased inpatient and outpatient healthcare utilisation costs.^7^

Cardiac rehabilitation (CR) is essential for the comprehensive management of patients with CVD, and is a multicomponent programme that targets secondary prevention and also improvement of patient’s psychosocial health.^8^ A recent Cochrane review has demonstrated that CR is effective^9^ and it reduces depressive symptoms.^10^ The assessment of depression is widely recommended by the recent clinical guidelines as it is a well-established risk factor for worse cardiac prognosis and outcomes.^3 8 11 12^

The time onset of depressive symptoms has been the focus point in the recent studies with regard to cardiac morbidity and mortality.^13–16^ Although some studies revealed that patients with history of depression prior to cardiac event experienced increased cardiac morbidity and mortality,^13 14^ other studies have found that patients with new-onset depressive symptoms after cardiac event were more likely to experience adverse cardiac events and mortality.^15 16^ A recent National Audit of Cardiac Rehabilitation (NACR)–based study has contributed to this literature and found that baseline characteristics of patients with history of depression such as higher anxiety, higher total number of comorbidities, smoking, physical inactivity and male gender were determinants of their depression levels following CR.^17^ Moreover, previous studies were unable to be inclusive of different types of comorbidities and their association with depression outcome in CR setting, and were also unable to adjust for heart failure (HF) and cardiac treatments. Therefore, the current study is the first to examine the impact of comorbidities and other patient characteristics on the depression outcome in the context of UK CR programmes.

## Methods

The strengthening the reporting of observational studies in epidemiology (STROBE) checklist was employed to report this study.^18^

### Data collection

Patient data based within NACR database were extracted and analysed. The primary aim of the NACR is to monitor CR programmes and improve the quality of delivery in UK CR centres and outcomes. To be able to achieve this, CR programmes collect individual patient data under section 251 approval of the NHS Act 2006 which is entered and secured in an online system held by NHS Digital. The identifiable patient data can be collected by the approval that NHS Digital has and these data are then anonymised before being made available for the NACR. Thus, due to this data governance process, there was no need to gain patient consent from each individual. NHS Digital reviews the data governance agreement between NACR and NHS Digital annually. In the present study, no additional NHS ethical approval was required because the data are used in line with NACR purposes and complies with data protection regulations. Currently, the number of CR services entering the data electronically is 229 which is 80% of all programmes.^19^ The data include patients’ initiating event, demographics, risk factors, treatment, medication and outcomes who undergo CR in the UK.

### Participants

The analysis was based and data extracted from the NACR between 1 April 2012 and 31 March 2018. The study population included patients with myocardial infarction (MI) and HF and those who receive treatment of percutaneous coronary intervention (PCI) and coronary artery bypass graft (CABG) as recommended in the clinical guidelines.^20 21^ All the eligible patients who did not present with prior history of depression and who had pre-HADS and post-HADS assessments recorded in CR (n=64 658) were selected as participants during the study period.

### Measures

Patients who had no reported prior history of depression and had valid pre-CR and post-CR HADS measurements were selected using the NACR data set, and with this approach, eligible patient population was defined for the study sample. History of depression in the NACR data is confirmed by CR practitioners with case note review and by patients if they have ever been diagnosed or treated by a doctor for depression.

### Hospital Anxiety and Depression Scale (HADS)

The HADS is an assessment tool in the form of a self-answered questionnaire which is employed for screening depressive symptoms in clinical practice. The HADS is one of the psychosocial health measurements that is recommended for assessment before and after CR to provide patients with tailored CR according to their needs.^8^ There are 14 items in HADS, 7 of which cover anxiety symptoms and 7 depressive symptoms. The score of 0 to 3 can be assigned to each item, accordingly a minimum of 0 and maximum of 21 can be received for the separate anxiety and depression scores, and the higher the scores the worse the symptom is. The HADS has been demonstrated to be a reliable and valid measure for assessing anxiety and depression measurements, and is therefore recommended to use with patients with CVD.^22–24^ In the current study, the analysis and the clinical cut-off point of 8 was used to categorise patients into patients with absence of new-onset depressive symptoms (<8) and presence of new-onset depressive symptoms (≥8) groups.^24^ The reason for this is that a systematic review has demonstrated that an optimal balance between sensitivity and specificity for HADS as a screening tool was often achieved at a cut-off score of 8 for both HADS anxiety and HADS depression, considering the specificity and sensitivity for both scales at roughly 0.80.^23^ The analysis used in the present study then compared the patients with HADS <8 and HADS ≥8 in a subgroup of patients without history of depression. In addition, baseline HADS anxiety scores that are routinely reported as part of HADS were employed to see whether it determines depression outcomes after CR.

### Total number of comorbidities and comorbidity types

Total number of comorbidities is defined as the sum of the number of comorbidities that the patients had including angina, diabetes, stroke, emphysema, chronic back problems and others. Comorbidities are defined as the medical history of conditions in the NACR data which is confirmed by CR providers with case note review and by patients answering the question of whether they have been diagnosed or been treated for the condition.

### Other variables

The patient demographics used in the analysis were age, gender and marital status (single/partnered). The English Index of Multiple Deprivation (IMD), a measure of deprivation used in England, was another patient demographic. Seven domains are applied to construct the IMD measure: employment, health deprivation and disability, income, crime, barriers to housing and services, living environment, education skills and training.^25^ In total, there are 32 844 sub-areas ranked from the most to least deprived areas. IMD was used to categorise patients into two quintiles where the first quintile, reported as ‘lowest quintile’, means the most deprived areas and other quintiles categorised as the less deprived. Pre-CR, baseline smoking measurements are categorised according to whether the patient was a current smoker or non-smoker. Other included variables were weight (kg), moderate physical activity (150 min a week), presence of heart failure (yes/no) and cardiac treatment (PCI/CABG/other/none). All the variables included in this paper have been chosen in line with the literature and baseline clinical assessment variables carried out by CR practitioners. These variables were explained in detail in previous publications.^17 26 27^

### Statistical analysis

The data analyses were performed using the IBM SPSS software statistics V.25. Patients without prior history of depression who have pre-HADS and post-HADS assessments recorded constituted the study population. A p value of <0.05 was considered to be statistically significant. Summary statistics were presented as means, SD and percentages. The descriptive statistics were calculated, and the baseline characteristics compared between patients with absence of new-onset depressive symptoms and presence of new-onset depressive symptoms, using t-tests for continuous variables and χ^2^ tests for categorical variables. Cohen’s d effect size was calculated for continuous variables and phi or Cramer’s V effect size was reported for categorical variables. A binary logistic regression model was conducted in order to investigate which variables determined improvement in the HADS depressive symptoms following CR.

## Results

**Figure 1:**
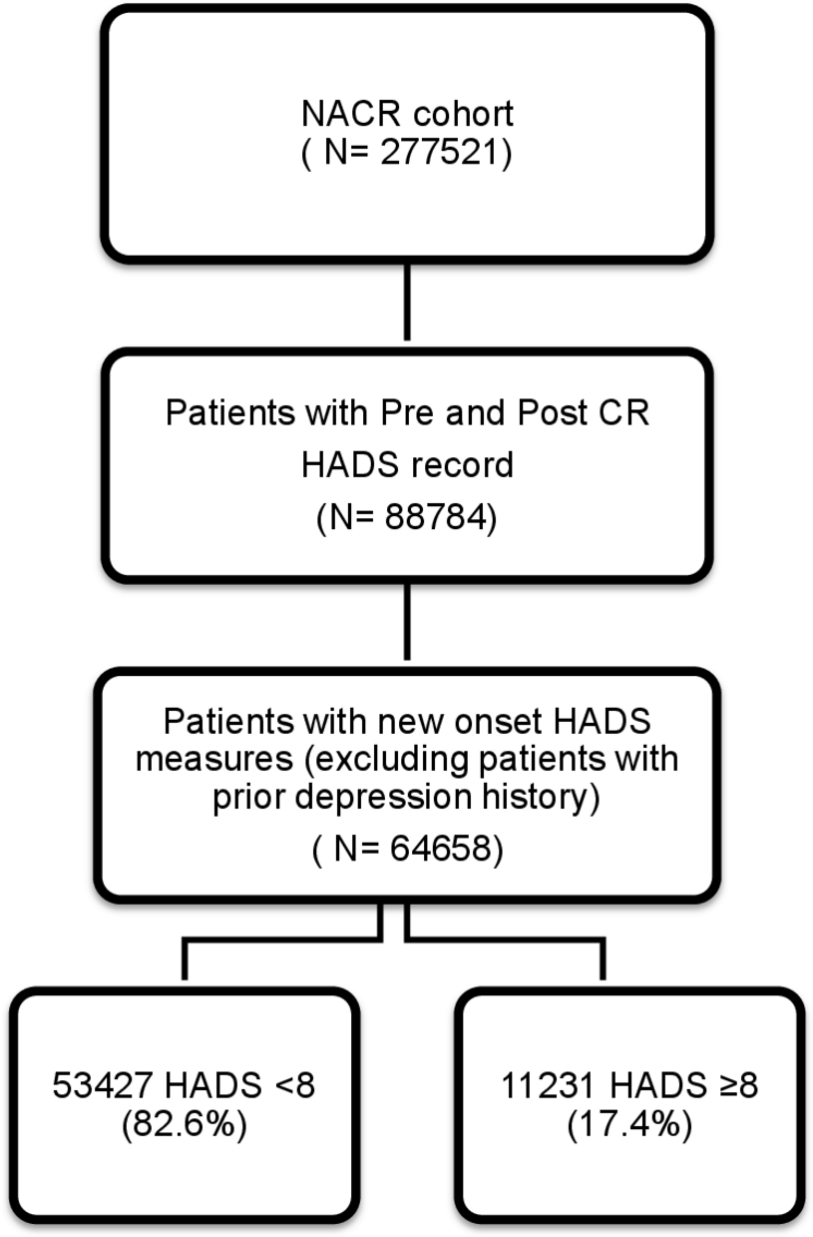
Study flow diagram. CR, cardiac rehabilitation; HADS, Hospital Anxiety and Depression Scale; NACR, National Audit of Cardiac Rehabilitation.

A total of 64 658 patients without prior history of depression who had completed CR with valid pre-HADS and post-HADS assessments constituted the study population. From this population of 64 658 participants, 17.4% presented with new-onset depressive symptoms (HADS ≥8) and 82.6% were, for this study, defined as having an absence of new-onset depressive symptoms (HADS <8). The total population during the study time period and the study sample size are shown in the flow diagram in figure 1. At CR baseline assessment, patients with new-onset depressive symptoms (HADS ≥8 group) differed from the HADS <8 group in terms of 11 characteristics in that they were younger, female, single, from areas of higher deprivation, had a higher total number of comorbidities, more weight, higher anxiety scores, physically inactive, more likely to smoke, presence of heart failure and less likely to receive cardiac treatments. The HADS ≥8 group presented with a higher proportion of comorbidities including angina, diabetes, stroke, emphysema, and chronic back problems compared with those with an HADS <8 group. Baseline characteristics of patients according to high and low HADS scores (HADS <8, absence of new-onset depressive symptoms; HADS ≥8, presence of new-onset depressive symptoms) are presented in table 1.

**Table 1:** Baseline characteristics for presence and absence of new onset HADS depressive symptoms groups

Variables	HADS <8 group (n=53 427)	HADS ≥8 group (n=11 231)	P value	Effect size
	Mean±SD	Mean±SD		
Age	66.56±10.54	64.73±11.29	<0.001	0.17
Total comorbidities	2.34±1.44	2.58±1.56	<0.001	0.16
Weight	82.35±16.46	83.11±18.40	<0.001	0.05
HADS anxiety score	4.38±3.31	9.56±4.03	<0.001	1.50
Gender female %	24.1	29.3	<0.001	0.05
150 min physical activity a week (yes) %	46.2	28.5	<0.001	0.14
Smoking (yes) %	4.7	8.3	<0.001	0.06
Single %	20.1	24.8	<0.001	0.04
IMD (most deprived) %	9.6	15.0	<0.001	0.07
Heart failure (yes) %	6.4	10.1	<0.001	0.06
Comorbidity				
Angina %	18.4	19.5	0.008	0.01
Diabetes %	19.1	24.6	<0.001	0.05
Stroke %	4.2	5.9	<0.001	0.03
Emphysema %	1.7	2.6	<0.001	0.03
Chronic back problems %	11.5	14.3	<0.001	0.03
Cardiac treatment				
No treatment %	8.5	11	<0.001	0.05
PCI %	50.3	44.5		
CABG %	16.6	16.2		
Other treatment %	24.6	28.3		

CABG, coronary artery bypass graft; HADS, Hospital Anxiety and Depression Scale; IMD, Index of Multiple Deprivation; PCI, percutaneous coronary intervention.

A binominal logistic regression was performed to ascertain the impact of the 11 patient characteristics and 5 identified comorbidities on the likelihood that participants’ depression symptoms improved between HADS depression symptom group following CR. The logistic regression model was statistically significant, χ^2^(18)=359.814, p<0.001. The model correctly classified 63.3% of the cases. Hosmer and Lemeshow test shows that the model was a good fit (p=0.208). Of the 16 predictor variables, 13 were statistically significant: weight, HADS anxiety score measurement, physical inactivity, smoking, marital status, total number of comorbidities, presence of heart failure, CABG or other treatments, comorbidities of angina, diabetes, stroke, emphysema and chronic back problems (as shown in table 2).

**Table 2:** Coefficients of the model determining change in depression whether a patient has improved depressive symptoms after CR

Variable	B	SE	P value	OR	Lower 95% CI	Upper 95% CI
Age	−0.005	0.003	0.138	0.995	0.990	1.001
Total no of comorbidities	−0.071	0.024	0.004	0.932	0.888	0.977
Weight	−0.008	0.002	<0.001	0.992	0.989	0.996
HADS anxiety score	−0.105	0.008	<0.001	0.900	0.885	0.915
150 min a week physical activity (no)	−0.196	0.067	0.003	0.822	0.721	0.937
Smoking (yes)	−0.281	0.118	0.018	0.755	0.599	0.952
Gender (male)	−0.119	0.075	0.112	0.888	0.767	1.028
Marital status (single)	−0.273	0.073	<0.001	0.761	0.660	0.877
IMD (most deprived)	−0.015	0.088	0.864	0.985	0.829	1.171
Angina (yes)	−0.251	0.083	0.003	0.778	0.661	0.916
Diabetes (yes)	−0.186	0.077	0.016	0.830	0.714	0.965
Stroke (yes)	−0.332	0.136	0.015	0.718	0.550	0.937
Emphysema (yes)	−0.407	0.207	0.049	0.665	0.443	0.999
Chronic back problems (yes)	−0.208	0.095	0.028	0.812	0.674	0.977
Heart failure (yes)	−0.289	0.105	0.006	0.749	0.610	0.919
Cardiac treatment (reference: no treatment)						
PCI	0.078	0.116	0.499	1.082	0.862	1.357
CABG	0.362	0.132	0.006	1.436	1.108	1.861
Other treatment	0.313	0.119	0.008	1.367	1.084	1.725
Constant	2.720	0.320	<0.001	–	–	–

B, regression coefficient; CABG, coronary artery bypass graft; CR, cardiac rehabilitation; HADS, Hospital Anxiety and Depression Scale; IMD, Index of Multiple Deprivation.

Patients with higher number of comorbidities had 0.932 times lower odds of residing in low HADS levels after CR. Increased HADS anxiety score was associated with a 10% decreased likelihood of improved depressive symptoms after CR. Physical inactivity was also associated with 18% reduced odds of moving to absence of HADS depressive symptoms category. Smoking was associated with 25% decreased likelihood of improvement in HADS range after CR. Increased weight and being single were also negative determinants of change in depression. Patients who have the comorbidity of emphysema had 0.665 times lower odds of having improved depressive symptoms which is followed by having the following conditions: comorbidity stroke, comorbidity angina, comorbidity chronic back problems and comorbidity diabetes (0.718, 0.778, 0.812 and 0.830 times, respectively). Patients who had heart failure were 25% less likely to improve their depressive symptoms following CR. In addition, patients who receive CABG and other treatments were 43% and 36% increased odds of having improved depressive symptoms.

## Discussion

Previously, the association of depressive symptoms with poor cardiac prognosis, cardiac mortality and overall mortality is well established in the literature. Yet, there remained a need for thorough investigation of the patient characteristics including demographics, comorbidities and clinical characteristics associated with new-onset depressive symptoms. Thus, the current study examined the characteristics of patients with new-onset depressive symptoms in CR patients to a greater extent. The findings of our study demonstrated that baseline characteristics of patients with new-onset depressive symptoms determine the change in the depression outcome after CR. More specifically, patient characteristics such as having a higher number of comorbidities and the comorbidities of angina, diabetes, stroke, emphysema, chronic back problems, increased weight, higher anxiety scores, physical inactivity, smoking, presence of heart failure, CABG treatment and being single were significant predictors of depression outcomes following CR. However, age, gender and IMD were not able to determine depressive symptoms after CR in patients with new-onset depressive symptoms.

A finding of note was that having a higher total number of comorbidities was a significant determinant of depressive symptoms. A prior study employing RCT data in their analysis was unable to find an association between comorbidities and depressive symptoms,^28^ which perhaps could be explained by the younger population (mean age 59.1±19.8) compared with the current study (66.24±10.69). Indeed, a recent meta-analysis of CR trials recommends future trials involve patients who are more representative of the broader patients with CVD, including patients with comorbidities.^9^ In addition, patients who present with multiple comorbidities are found to be less likely to be referred to or uptake CR which is a primary challenge for healthcare providers and CR programmes.^29 30^ Yet, attending CR enables patients with multiple comorbidities to benefit in terms of improving their functional capacity and psychosocial conditions.^30 31^

Another finding was that the comorbidity of diabetes was associated with 17% reduced likelihood of improvement in depressive symptoms following CR (OR 0.830 95% CI 0.714 to 0.965). Patients with diabetes attending CR had reduced physical fitness and increased cardiac risk factors compared with non-diabetic patients.^32^ Individuals with diabetes who participate or complete CR were also significantly less likely than non-participants or no-completers to experience mortality.^33 34^ When the condition of patients with diabetes was taken into account in terms of having a greater cardiac risk profile and lower programme participation rate, recommendations have been made to CR programmes to target patients with diabetes and involve them in CR.^35–37^ In addition, considering the prevalence of diabetes has been steadily increasing over time,^38^ the medical management of diabetes may relatively be necessary and perhaps can reduce depressive symptoms.

The stroke comorbidity was influential on the depression outcome in patients with new-onset depressive symptoms after CR. It was associated with reduced odds of improvement in depressive symptoms following CR (OR 0.718, 95% CI 0.550 to 0.937). Previous studies have shown that patients with stroke comorbidity were less likely to be referred to^29^ and uptake CR.^30^ However, the study of Marzolini *et al*^39^ found that patients who had a stroke who undergo CR improve their functional capacity and cardiovascular fitness; for that reason, including patients who had a stroke in CR can be beneficial.

Emphysema is the comorbidity which had the highest impact, among the comorbidities, on the change in depressive symptoms following CR. Having emphysema at baseline was associated with 34% reduced odds of improvement in the depressive symptoms after CR. Patients with chronic obstructive pulmonary disease (COPD) and CVD experience problems of breathlessness and disability in the context of multi-morbidity, thus, cardiac rehabilitation services are recommended to take positive action by providing tailored intervention options for patients with COPD.^40^ In addition, angina and chronic back problems were other comorbidities that were negative determinants of depression outcomes following CR with OR 0.778, 95% CI 0.661 to 0.916 and OR 0.812, 95% CI 0.674 to 0.977, respectively. Overall, all of the comorbidities that were mentioned were negative determinants of improvement in CR patient’s depressive symptoms following CR in patients who present with new-onset depressive symptoms. This is the first study showing the influence of the variety of comorbidities on CR outcomes in patients with new-onset depressive symptoms.

Our finding of clinical relevance was that smoking, physical inactivity and weight were modifiable CVD risk factors having an unfavourable impact on depression outcomes after CR. These factors were negative determinants of improvement in depression in patients with new-onset depressive symptoms following CR (OR 0.755, 95% CI 0.599 to 0.952; OR 0.822, 95% CI 0.721 to 0.937; OR 0.992, 95% CI 0.989 to 0.996, respectively). These results were in line with previous systematic reviews conducted in the general population,^41–43^ and other cohort studies that involved patients with CVD.^44 45^ However, these studies were unable to include CR patients and specifically patients with new-onset depressive symptoms. The current study, on the other hand, included patients who undergo CR and assessed outcome of change in depressive symptoms following CR. Prior studies have tended to focus on CR utilisation and depression with little in the way of evidence regarding factors determining outcome for patients with new-onset depression.

Another finding of the current study is patients with new-onset depressive symptoms and who have higher anxiety score at baseline were less likely to improve their depression symptoms (OR 0.900, 95% CI 0.885 to 0.915). This finding demonstrates that anxiety and depression are interrelated and associated with poor outcomes which was in accordance with a previous study.^46^ In the USA-based study, 622 patients with HF were included and it was revealed that anxiety, measured by Brief Symptoms Inventory, was associated with depressive symptoms in both men and women. In our study, HADS anxiety score measurement was employed for the assessment instead and still confirmed this association. In addition, the current study is first to show that patients with HF were 25% less likely to improve their depressive symptoms following CR. This result, perhaps, may explain why a recent CR trial was unable to demonstrate improvement in depressive symptoms in HF population.^47^ Thus, patients with HF may need additional psychosocial support from CR teams which may require further investigation in future studies. Moreover, the cardiac treatment was also adjusted in the multivariate analysis in the current study and it has been found that patients who receive the treatment of CABG were 43% more likely to have improved depressive symptoms.

In the current study, considering patient demographics, being single was associated with 24% reduced likelihood of improved depressive symptoms after CR (OR 0.761, 95% CI 0.660 to 0.877). Having partner support may improve patients’ coping with their illness and thereby improve their depression symptoms. The association of being single with increased depressive symptoms has also been found in meta-analysis of 24 cross-sectional and 8 longitudinal studies conducted by Yan *et al*.^48^ This study included 52 803 individuals from the general population of people aged ≥55 years. In our study, other patient demographics of age, gender and IMD were unable to significantly predict the depression outcome. In the multivariate analysis, the adjustments were made for other cardiac risk factors and comorbidities which may have an impact on this result. Bachmann *et al*^49^ recently found an association between lower neighbourhood socioeconomic context and reduced likelihood of participation in CR. In addition, patients who live in socially deprived areas were more disadvantaged than ones who live in less deprived neighbourhoods in terms of inhabiting poor health behaviours such as smoking and being physically inactive.^50^ In the current study, although the IMD was found to be associated with depressive symptoms at baseline assessment, after adjusting for other covariates it has no longer reached statistical significance.

Finally, the model of logistic regression was chosen to match with other literature in this field; for comparisons to be made, future work may look to invoke a propensity-based linear model of change accounting for included and non-included variables.

### Limitations

In order to examine the determinants of outcome, specifically in patients with new-onset depressive symptoms, patients with prior history of depression have been excluded from our population. However, looking at the characteristics of our sample, it was representative of all available patients during the study time scale (n=277 521), mean age was 66.24 compared with 65.06, 25% female compared with 27%, and other variables did not differ by more than 4%. The sample was nationally representative of patients with new-onset depressive symptoms in the UK, yet, it is important to state that not all CR programmes provide a full record of patients who complete CR and in fact, in NACR data, 36.6% did not have follow-up assessment which may have an influence on the representativeness of the sample.^19^ A strength of this study was the use of an observational approach by employing analysis of routinely collected clinical data which enabled us to generate real-world understanding. In addition, the data involved more patients with multi-comorbidities and higher female percentage than prior RCTs.^9^ However, as this is a registry-based study, it should be noted that observational studies do not allow to draw causal conclusions, only association. The analysis was not able to take account of the treatment with antidepressant medication or diagnosis of depression in the CR period, as this was not recorded in the NACR data set.

## Conclusion

The primary objective of this study was to ascertain whether comorbidities, demographic and clinical characteristics of patients with new-onset post-cardiac event depressive symptoms determine improvement in their depression following CR. Baseline characteristics of patients with new-onset depressive symptoms such as comorbid conditions of angina, diabetes, stroke, emphysema and chronic back problems, as well as higher total number of comorbidities, increased weight, physical inactivity, smoking, presence of heart failure and being single were negative determinants of improvement in depression following CR. However, receiving CABG or other treatments were positive determinants of improvement. Our findings could promote CR programmes that focus on tailoring the CR intervention around comorbidity, physical activity status, weight management and smoking cessation in patients with new-onset depressive symptoms.
